# A single supratherapeutic dose of ridaforolimus does not prolong the QTc interval in patients with advanced cancer

**DOI:** 10.1007/s00280-012-1942-7

**Published:** 2012-08-10

**Authors:** Richard M. Lush, Amita Patnaik, Daniel Sullivan, Kyriakos P. Papadopoulos, Michele Trucksis, Jacqueline McCrea, Kristine Cerchio, Xiaodong Li, Mark Stroh, Diana Selverian, Keith Orford, Scot Ebbinghaus, Nancy Agrawal, Marian Iwamoto, John A. Wagner, Anthony Tolcher

**Affiliations:** 1 H. Lee Moffitt Cancer Center and Research Institute, 12902 Magnolia Drive, Tampa, FL 33612 USA; 2 South Texas Accelerated Research Therapeutics (START) Center for Cancer Care, San Antonio, TX USA; 3 Merck Sharp & Dohme Corp., Whitehouse Station, NJ USA

**Keywords:** Ridaforolimus, mTOR inhibitor, QTc interval, Safety

## Abstract

**Purpose:**

This dedicated QTc study was designed to evaluate the effect of the mammalian target of rapamycin inhibitor, ridaforolimus, on the QTc interval in patients with advanced malignancies.

**Methods:**

We conducted a fixed-sequence, single-blind, placebo-controlled study. Patients (*n* = 23) received placebo on day 1 and a single 100-mg oral dose of ridaforolimus on day 2 in the fasted state. Holter electrocardiogram (ECG) monitoring was performed for 24 h after each treatment, and blood ridaforolimus concentrations were measured for 24 h after dosing. The ECGs were interpreted in a blinded fashion, and the QT interval was corrected using Fridericia’s formula (QTcF). After a washout of at least 5 days, 22 patients went on to receive a therapeutic regimen of ridaforolimus (40 mg orally once daily for 5 days per week).

**Results:**

The upper limit of the two-sided 90 % confidence interval for the placebo-adjusted mean change from baseline in QTcF was <10 ms at each time point. No patient had a QTcF change from baseline >30 ms or QTcF interval >480 ms. Geometric mean exposure to ridaforolimus after the single 100-mg dose was comparable to previous experience with the therapeutic regimen. There appeared to be no clear relationship between individual QTcF change from baseline and ridaforolimus blood concentrations. Ridaforolimus was generally well tolerated, with adverse events consistent with prior studies.

**Conclusions:**

Administration of the single 100-mg dose of ridaforolimus did not cause a clinically meaningful prolongation of QTcF, suggesting that patients treated with ridaforolimus have a low likelihood of delayed ventricular repolarization.

**Electronic supplementary material:**

The online version of this article (doi:10.1007/s00280-012-1942-7) contains supplementary material, which is available to authorized users.

## Introduction

Ridaforolimus (AP23573, MK-8669) is a specific inhibitor of the mammalian target of rapamycin (mTOR), a serine/threonine kinase that has a key role in integrating intracellular signals necessary for cell growth, metabolism, and survival [6, 15]. The activity of mTOR is normally regulated by receptor tyrosine kinases that activate the phosphatidylinositol-3-kinase (PI3K)/Akt pathway [2, 5]. Intracellular signaling in the PI3K/Akt pathway is dysregulated in many malignancies, usually through gene mutation or overexpression of key pathway components or regulatory factors, which leads to increased mTOR activity [2, 24]. As a result, mTOR represents an attractive therapeutic target in cancer.

Preclinical studies have shown that ridaforolimus has antiproliferative activity against a broad range of human tumor cell lines in vitro and in tumor xenograft models in vivo [21]. Ridaforolimus has also displayed promising activity in phase 1 and phase 2 clinical trials in patients with advanced sarcoma and other malignancies [11, 17, 20, 22]. In the recently completed phase 3 Sarcoma mUlti-Center Clinical Evaluation of the Efficacy of riDaforolimus (SUCCEED) trial, maintenance therapy with ridaforolimus administered at a dose of 40 mg orally once daily for 5 consecutive days every week significantly improved progression-free survival compared with placebo, in advanced sarcoma patients who achieved clinical benefit (complete response, partial response, or stable disease) from prior cytotoxic chemotherapy [4].

Assessment of the potential for corrected QT interval (QTc) prolongation is an essential component of new drug development that was prompted by deaths attributed to cardiac arrhythmias with certain drugs such as terfenadine and cisapride [7, 23]. The International Conference on Harmonisation (ICH) formulated the E14 guidance document, endorsed by the US Food and Drug Administration, which specifies that a dedicated QTc study should be performed for all new drugs in order to evaluate the risk of QTc prolongation as a biomarker for ventricular tachycardias, especially torsade de pointes [3, 7, 9]. Electrocardiogram (ECG) monitoring during phase 1 and phase 2 clinical trials of ridaforolimus did not identify a risk of QTc prolongation. However, these clinical studies are generally considered not to accurately determine the true risk of QTc prolongation with ridaforolimus because ECG measurements were not scheduled to coincide with clinically relevant pharmacokinetic time points (i.e., the time of maximum ridaforolimus blood concentrations) and the doses were not sufficient for evaluating whether ridaforolimus affects QTc.

The present QTc study was specifically designed to evaluate the effect of a single 100-mg oral dose of ridaforolimus on the QTc interval in advanced cancer patients. This study was developed using the recommendations in the ICH E14. Conducting this study in cancer patients placed some notable restrictions on the study design; however, many of the recommended critical ICH components were incorporated, resulting in a dedicated, robust study design evaluating the potential effect of an anticancer agent (not amenable for study in the healthy volunteer population) on ventricular repolarization [19].

## Methods

### Study design

This 2-part, phase 1 study was conducted at 2 US centers (ClinicalTrials.gov identifier: NCT00874731; http://clinicaltrials.gov/ct2/show/NCT00874731; Protocol 037). Part 1 had a fixed-sequence, single-blind, placebo-controlled design consisting of 2 days of intensive ECG monitoring for QTc assessment. Patients received a single oral dose of placebo on day 1 and a single 100-mg oral dose of ridaforolimus on day 2 while sequestered at the investigational site. During this part of the study, treatment was administered in a fasted state, with fasting continued until 4 h postdose and water restricted for 1 h before and after dosing. Because the study population had advanced malignancies that were not amenable to standard anticancer therapy, patients were offered the opportunity to receive ridaforolimus in a standard therapeutic regimen after completing the QTc evaluation. The optional part 2 of the study had an open-label design, with clinic visits scheduled every 28 days; it was separated from part 1 by a washout period of at least 5 days. Patients received ridaforolimus 40 mg once daily for 5 consecutive days every week until disease progression, unacceptable toxicity, or withdrawal from the study. A poststudy assessment was scheduled approximately 30 days after the last dose of study treatment or before initiation of any new treatment.

The study was conducted in compliance with Good Clinical Practice standards and regulatory requirements for ethical committee review, informed consent, and protection of human subjects participating in biomedical research. All subjects provided written informed consent before any study-related procedures were conducted.

### Patients

Men and women at least 18 years old were eligible if they had a histologically or cytologically confirmed metastatic or locally advanced malignancy that progressed after standard therapy or for which no standard therapy exists. There was no limit on the number of prior treatment regimens. Eligible patients had Eastern Cooperative Oncology Group performance status of 0–2, adequate hematologic, renal, and hepatic functions, coagulation parameters ≤1.2 times the upper limit of normal, serum potassium and magnesium within normal limits, and a life expectancy of >3 months. Females of childbearing potential agreed to use 2 approved contraceptive methods from screening until 30 days after the last dose of ridaforolimus; males with a female partner of childbearing potential also agreed to use medically acceptable contraception during this time period.

Patients were excluded if they had received chemotherapy, radiotherapy, or biological agents within 4 weeks of the first dose of treatment (6 weeks for monoclonal antibodies, nitrosoureas, or mitomycin C), had not recovered from adverse events (AEs) due to prior therapy, or were receiving concurrent anticancer therapy (except luteinizing hormone–releasing hormone analogs for prostate cancer or supportive therapy) or immunosuppressive therapy (except stable doses of corticosteroid replacement therapy). Patients with specific ECG intervals (PR >0.26 s, QRS ≥0.12 s, Fridericia-corrected QT [QTcF] ≥470 milliseconds (ms), RR >1.2 s, or ventricular rate <50 beats per minute after sitting quietly for 10 min), history of risk factors for torsade de pointes (e.g., heart failure, uncorrected hypokalemia, family history of long QT syndrome), or a history of sick sinus syndrome, second- or third-degree atrioventricular block, myocardial infarction, unstable angina pectoris, or cardiac arrhythmia were also excluded. Other exclusion criteria were primary central nervous system tumor or active brain metastases, newly diagnosed or poorly controlled diabetes, recent history of drug or alcohol abuse, human immunodeficiency virus positive or known history of hepatitis B or C, treatment with medications that induce or inhibit cytochrome P450 (CYP3A4) within 2 weeks, treatment with medications known to prolong QTc interval within 4 weeks, active infection or intravenous (IV) treatment with antimicrobial agents within 2 weeks, prior high-dose chemotherapy with stem-cell rescue, blood transfusion within 1 week, or participation in a study of an investigational compound or device within 30 days.

### Electrocardiogram assessments

Holter ECG monitoring was performed for 24 h after each dose in part 1 using a Mortara H12+ digital Holter recorder. Patients rested in a supine position for at least 10 min before and 5 min after each prespecified ECG time point (predose and at 0.5, 1, 2, 3, 4, 6, 8, 10, and 24 h after dosing). Five ECG recordings were extracted from the Holter monitor at each of these time points, according to a prespecified algorithm at a centralized ECG core laboratory (Quintiles ECG Services; Mumbai, India). The ECGs were interpreted by cardiologists who were blinded to treatment allocation, patient, and time of the ECG. The ECG recordings for a single subject were evaluated by a single cardiologist reader on the same day. At each time point, the ECG intervals in the 5 recordings were averaged to reduce variability and increase precision of the estimate. QT interval was measured in lead II, with an alternate lead used if the lead II recordings were of suboptimal quality, and Fridericia’s correction to the QT interval (QTcF = QT/RR^0.33^) was made in order to correct for heart rate [10].

### Pharmacokinetic analysis

Ridaforolimus blood concentrations were determined on day 2 during the first part of the study. Blood samples (5 mL) were collected before dosing and at 0.5, 1, 2, 3, 4, 6, 8, 10, and 24 h after dosing. Samples were analyzed for blood ridaforolimus concentrations by Charles River Laboratories Preclinical Services (Shrewsbury, MA) using a validated high-performance liquid chromatography/mass spectrometry/mass spectrometry (HPLC–MS/MS) method after isolation of the analyte by liquid–liquid extraction [11]. The lower limit of quantitation for the method was 0.2 ng/mL, with a linear calibration range of 0.2 ng/mL to 400 ng/mL. Pharmacokinetic parameters were determined by standard methods: maximum blood concentration (*C*_max_) and time to maximum concentration (*T*_max_) were obtained by inspection of the blood concentration data, and area under the concentration–time curve from zero to 24 h (AUC_0-24_) was calculated using the linear trapezoidal rule for ascending concentrations and the logarithmic trapezoidal rule for descending concentrations. Plasma samples were collected, but not assayed; this is because ridaforolimus disproportionately partitions into red blood cells and is highly bound to proteins.

### Safety assessments

Safety was assessed through clinical and laboratory evaluations while patients were sequestered at the investigational site during part 1, at clinic visits every 28 days during part 2, and at the poststudy visit. Vital signs were measured after patients rested in a semirecumbent position for 10 min predose, at 4 and 24 h after dosing in part 1, and at each subsequent clinic visit. Standard 12-lead ECG recordings were also obtained, including 8 h postdose in part 1. Cardiac troponin I levels were determined as part of the laboratory assessment in part 1 because preclinical safety studies had demonstrated a small risk of cardiac myonecrosis in primates. All AEs were assessed in terms of relationship to study treatment, and their intensity was graded by the investigator using the National Cancer Institute’s Common Terminology Criteria for Adverse Events (CTCAE version 3.0). Clinical AEs were classified using the Medical Dictionary for Regulatory Activities (MedDRA version 11.0)–preferred terms by system organ class.

### Statistical analysis

The primary end point of the study was the placebo-corrected change in QTcF from baseline, with the baseline value for each patient defined as the average of 5 replicate QTcF measurements made predose. The change from baseline in QTcF was analyzed using a repeated-measures mixed model, with treatment, time, and treatment-by-time interaction as fixed factors and subject as the random factor. The mean of the difference in QTcF change from baseline between ridaforolimus and placebo and its upper limit of the two-sided 90 % confidence intervals (CIs), equivalent to an upper one-sided 95 % CI limit, were calculated at each prespecified time point using the appropriate error term from the model and referencing a* t*-distribution. The incidence of QTcF values ≤450, >450, >480, and >500 ms and changes from baseline in QTcF <30, ≥30, and ≥60 ms were summarized by treatment and time point. Changes from baseline in QTcF versus ridaforolimus blood concentration were evaluated graphically using individual patient data. Since there was no clinically meaningful QTc prolongation based on the formal statistical analysis of placebo-corrected changes from baseline, no formal pharmacokinetic/QTc modeling was performed. Safety and pharmacokinetic parameters were tabulated and summarized using descriptive statistics.

## Results

### Patient disposition, demographics, and baseline characteristics

Twenty-three patients were enrolled in the study: 15 women and 8 men. The mean age was 54.4 years, and most patients were white (95.7 %) (Table 1). The most common malignancies were bone and soft tissue sarcoma (39.1 %) and colon cancer (21.7 %). The cohort was heavily pretreated: all patients had received at least 2 lines of prior chemotherapy, with a mean of 4.4 prior regimens. Most patients (91.3 %) received concomitant medications during part 1 of the study—most frequently analgesics (60.9 %), mineral supplements (39.1 %), and vitamins (39.1 %). All medications known to affect the CYP3A4 metabolism of ridaforolimus were discontinued before the study (except for one patient who continued to take pioglitazone).

**Table 1:** Demographics and baseline characteristics

Characteristic	Number of patients (*N* = 23)
Gender, *n* (%)	
Male	8 (34.8)
Female	15 (62.5)
Age, years	
Mean (SD)	54.4 (11.6)
Range	26.0–75.0
Race, *n* (%)	
Black/African-American	1 (4.3)
White	22 (95.7)
Cancer type, *n* (%)	
Bone and soft tissue sarcoma	9 (39.1)
Breast	3 (13.0)
Colon	5 (21.7)
Other (e.g., cervical, esophageal, non–small cell lung, rectal, ureteral, and uterine sarcoma)	6 (26.1)
Prior regimens	
Mean, *n* (SD)	4.4 (2.1)
Range	2.0–9.0

*SD* standard deviation

All patients (*N* = 23) who were enrolled in the study received placebo on day 1. Following placebo treatment, QTcF data were available for 22 patients; one patient did not have QTcF data since the QT interval could not be measured in one patient due to nonspecific T wave changes. One patient discontinued due to disease progression after receiving placebo on day 1 of part 1. Twenty-two patients received ridaforolimus 100 mg on day 2 of part 1 and subsequently entered part 2 of the study. After ridaforolimus treatment on day 2, QTcF data were available for 20 patients. One patient could not be evaluated (the same patient previously excluded from day 1 QTcF data). The other patient took a protocol-violating medication (CYP3A4 inducer pioglitazone); however, for day 1 (placebo day), it was determined that this medication did not preclude the patient’s Holter data from being included, since ridaforolimus was not administered on day 1. Pharmacokinetic data were available for 21 patients, as 2 patients were not included (the one patient who discontinued and the other patient who took a protocol-violating medication).

### Electrocardiogram and pharmacokinetic effects

**Fig. 1:**
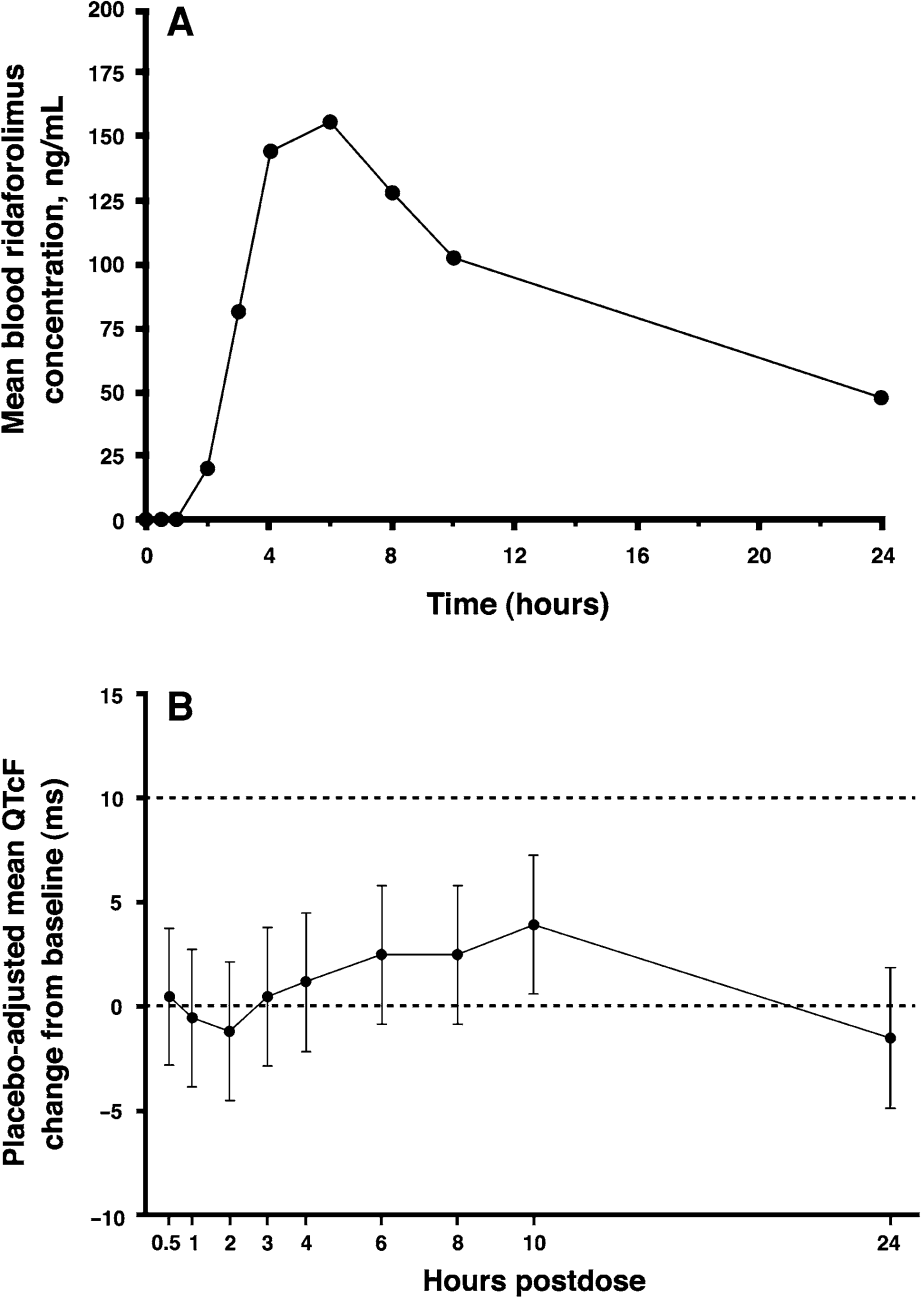
**a** Arithmetic mean blood concentration–time profile after oral administration of single 100-mg dose of ridaforolimus to patients with advanced cancer (*n* = 21). One patient was excluded from average blood concentration profile due to a protocol violation. **b** Placebo-adjusted means and 90 % confidence intervals (CIs) for change from baseline of Fridericia-corrected QTc (QTcF) intervals. The upper limit of the 90 % CI for the placebo-adjusted mean change from baseline in QTcF was <10 ms at each time point, indicating that ridaforolimus did not prolong the QTcF interval

The average blood ridaforolimus concentration profile rose to peak levels over 4–6 h postdose and then decreased in an approximately biphasic manner until 24 h after oral administration of a single 100-mg dose (Fig. 1a). The geometric mean *C*_max_ of ridaforolimus was 186 ng/mL (95 % CI: 159, 218), and the median *T*_max_ was 4.2 h after dosing (Table 2). Administration of the single 100-mg dose of ridaforolimus did not prolong the QTcF interval, inasmuch as the upper limit of the 90 % CI for the placebo-adjusted mean change from baseline difference (between ridaforolimus and placebo) in QTcF was <10 ms at each time point (Fig. 1b; Online Resource 1). The largest placebo-corrected change from baseline occurred 10 h after dosing, with a mean difference of 3.89 ms (90 % CI: 0.60, 7.17) in QTcF change from baseline after ridaforolimus versus placebo administration. For all other time points, the 90 % CI included zero. Similar results were obtained in an exploratory analysis, in which the mean change from baseline in QTcF between ridaforolimus and placebo and its 90 % CIs were computed based on paired *t* tests.

**Table 2:** Summary statistics for ridaforolimus blood pharmacokinetic parameters after administration of a single 100-mg oral dose of ridaforolimus to advanced cancer patients

Pharmacokinetic parameter	Geometric mean (95 % CI) (*n* = 21)
AUC_0-24_ (h·ng/mL)	1,875 (1,623, 2,167)
*C*_max_ (ng/mL)	186 (159, 218)
*T*_max_ (h)^a^	4.2 (2.2, 10.0)

*CI* confidence interval, *AUC*_0-24_ area under the concentration–time curve from zero to 24 h, *C*_max_ maximum concentration, *T*_max_ time to maximum concentration

^a^Median (minimum, maximum) for *T*_max_

**Fig. 2:**
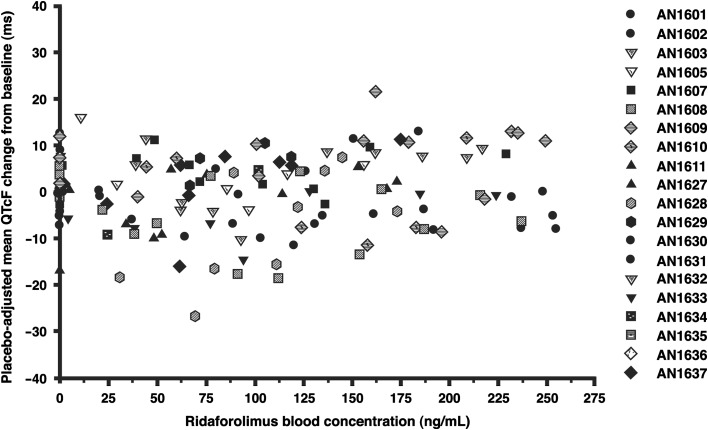
Individual QTc change from baseline versus ridaforolimus blood concentration after oral administration of single 100-mg dose of ridaforolimus to patients with advanced cancer. No clear relationship was evident between the individual Fridericia-corrected QTc (QTcF) changes from baseline and ridaforolimus blood concentrations

In the categorical analyses, none of the patients had an observed QTcF change from baseline >30 ms, and only one patient experienced a QTc interval >450 ms. This patient had day 1 QTcF values of 459.8, 453.8, 454.6, 450.4, and 451.2 ms at predose, 0.5 h after placebo, 1 h after placebo, 2 h after placebo, and 3 h after placebo, respectively, and values of 453.6 and 456.4 ms at 1 and 3 h after ridaforolimus on day 2, respectively. For the study cohort, no clear relationship was evident between the individual QTcF changes from baseline and ridaforolimus blood concentrations (Fig. 2). The appropriateness of using QTcF was evaluated graphically and by simple linear regression of QTcF versus RR interval using the placebo data: the estimated slope from the regression was 0.0060 (95 % CI: −0.0069, 0.0190), suggesting that Fridericia’s correction method was adequate.

Exploratory analyses were conducted on other ECG parameters. Mean PR and QRS intervals remained essentially unchanged during the 24-h periods after placebo and ridaforolimus; the largest placebo-corrected mean changes from baseline in PR and QRS intervals were 5.29 ms (90 % CI: 1.83, 8.74) and 3.47 ms (90 % CI: 2.13, 4.80), respectively, both seen at 24 h after dosing. In contrast, the mean RR interval varied during the 24-h dosing interval on days 1 and 2, with placebo-corrected values being negative at all time points; the largest placebo-corrected mean change from baseline in RR interval was −64.16 ms (90 % CI: −97.52, −30.80), which was observed at 24 h after dosing.

### Safety and tolerability

All 23 patients enrolled were evaluated for safety in part 1 after receiving placebo; the 22 patients who received ridaforolimus in part 1 of the study and subsequently entered part 2 were included in the safety evaluation. In part 1, a single 100-mg dose of ridaforolimus was generally well tolerated by patients with advanced cancer; no patient discontinued treatment due to an AE. Eleven patients (47.8 %) had AEs after placebo, and 18 patients (81.8 %) had AEs after the 100-mg dose of ridaforolimus. The most common AEs occurring in at least 15 % of patients were fatigue (27.3 %), thrombocytopenia (22.7 %), leukopenia (18.2 %), lymphopenia (18.2 %), and stomatitis (18.2 %). Although treatment was not blinded, 12 patients (54.5 %) had treatment-related AEs according to the investigator, most frequently thrombocytopenia and stomatitis; all treatment-related AEs were grade 1 or 2 (Table 3). Cardiac troponin I values remained within normal limits (0–0.78 ng/mL) in all patients.

**Table 3:** Treatment-related adverse events occurring in over 5 % of patients and all grade 3 events reported in any treatment group

Adverse event,* n* (%)^a^	Single 100-mg dose oral ridaforolimus (*n* = 22)		40-mg dose, once daily for 5 days/week (*n* = 22)	
	All grades	Grade 3	All grades	Grade 3
Leukopenia	3 (13.6)	0	1 (4.5)	0
Lymphopenia	3 (13.6)	0	2 (9.1)	1 (4.5)
Neutropenia	2 (9.1)	0	1 (4.5)	0
Thrombocytopenia	5 (22.7)	0	4 (18.2)	2 (9.1)
Diarrhea	0	0	6 (27.3)	0
Nausea	2 (9.1)	0	2 (9.1)	0
Stomatitis	4 (18.2)	0	6 (27.3)	0
Fatigue	1 (4.5)	0	6 (27.3)	2 (9.1)
Mucosal inflammation	0	0	8 (36.4)	1 (4.5)
Decreased appetite	1 (4.5)	0	3 (13.6)	0
Dysgeusia	1 (4.5)	0	2 (9.1)	0
Acne	0	0	2 (9.1)	0

^a^None of the patients who received placebo treatment (*n* = 23) experienced a treatment-related adverse event; no patient experienced events greater than grade 3 in any treatment group

In part 2 of the study, patients received a once-daily, 40-mg dose of ridaforolimus for 5 days every week for a median of 4.0 weeks (range 0.2–24.0 weeks; mean ± standard deviation: 6.7 ± 5.8 weeks). Ridaforolimus administered in part 2 was also generally well tolerated. Adverse events regardless of causality were experienced by 21 patients (95.5 %); the most common were mucosal inflammation or mucositis (40.9 %), fatigue (40.9 %), diarrhea (36.4 %), stomatitis (27.3 %), and decreased appetite (22.7 %). Most AEs were grade 1 or 2, did not require special attention, and were manageable with temporary dose reduction or supportive care measures. Treatment-related AEs were reported for 17 patients (77.3 %), most frequently mucosal inflammation or mucositis (36.4 %), stomatitis (27.3 %), fatigue (27.3 %), diarrhea (27.3 %), and thrombocytopenia (18.2 %). Thrombocytopenia and fatigue (each in 2 patients; 9.1 %) were the most common grade 3 events; no grade 4 events were reported. Five patients (22.7 %) required dose modifications until resolution or improvement of treatment-related AEs.

Serious AEs were reported in 8 patients (36.4 %), including 2 (9.1 %) with events considered related to treatment (viral bronchitis and pneumonitis). Three patients discontinued due to AEs: one patient discontinued due to treatment-related mucositis and 2 patients discontinued due to AEs unrelated to study treatment (elevated bilirubin and pneumonia). Two patients died during the course of the study due to disease progression. Laboratory safety testing revealed some clinically significant laboratory abnormalities; most notable was elevated uric acid levels experienced by 6 patients (26.1 %). Elevated uric acid had no physiologic consequences, and therefore, these were considered grade 1 events according to CTCAE criteria. Four patients (17.4 %) had elevated glucose, which is known to be associated with mTOR inhibition. Other safety assessments, including vital signs, physical examinations, and 12-lead ECGs, did not show clinically meaningful findings as a function of treatment.

## Discussion

The results of this dedicated QTc study demonstrate that administration of a single 100-mg oral dose of ridaforolimus does not prolong the QTcF interval in patients with advanced malignancies. The upper bound of the 90 % CI of the placebo-corrected mean QTcF change from baseline was <10 ms at every time point measured during the 24-h evaluation period. The categorical analyses of QTcF and change from baseline in QTcF further support the conclusion that ridaforolimus does not prolong QTcF. Only one patient had a QTcF interval >450 ms, which was observed after both placebo and ridaforolimus; no patient had a QTcF >480 ms or change from baseline >30 ms. Whole-blood pharmacokinetics of ridaforolimus were also determined over the 24-h period after dosing. The timing of blood collection coincided with the timing of ECG measurement in order to evaluate whether there was a concentration–time relationship, as recommended in E14 guidelines [13]. Individual QTcF changes from baseline versus ridaforolimus blood concentrations revealed no clear concentration–time relationship. Moreover, maximum exposure to ridaforolimus was observed 4–6 h after administration; at these time points, the placebo-corrected changes from baseline in QTcF were 1.18 ms (90 % CI: −2.10, 4.47) and 2.49 ms (90 % CI: −0.79, 5.78), respectively. These findings suggest that ridaforolimus is not likely to cause a clinically meaningful prolongation of the QTc interval in patients with cancer.

Since this study evaluated ridaforolimus in an advanced cancer population, its design was modified from the thorough QT/QTc study recommended in E14 guidance. A positive control that prolongs QTc was not included due to the overall poor health of the study population. A randomized crossover design was not used because the long half-life of ridaforolimus (~50 h) would have necessitated a long washout period, which would not have been ethical or acceptable for this population of advanced cancer patients. However, the study design did incorporate many key E14 recommendations, including the use of replicate ECG recordings to reduce variability, use of a centralized core laboratory blinded to time and treatment to reduce bias and variability, use of a placebo, and measurement of blood ridaforolimus concentrations at times of the ECG assessments to evaluate potential pharmacokinetic–pharmacodynamic relationships. A similar study design was used previously to evaluate the effect of vorinostat on QTc in advanced cancer patients [19].

The single 100-mg oral dose used in this study was selected for several reasons. First, this single supratherapeutic dose provided the highest attainable whole-blood ridaforolimus *C*_max_, given the toxicity limitations associated with administration of multiple supratherapeutic doses. The therapeutic dose of 40-mg oral ridaforolimus administered once daily for 5 consecutive days every week is the maximum tolerated dose; the onset of dose-limiting toxicity occurs within the first week at higher doses [17]. Second, the 100-mg dose is the highest single oral dose that has been administered to patients and was predicted by pharmacokinetic modeling to provide exposure at least comparable with that achieved with the therapeutic dose. Similar to other rapamycin analogs [12, 14, 16], ridaforolimus exhibits saturable binding to erythrocytes, which may be an important contributor to the drug’s nonlinear whole-blood pharmacokinetics, with less than proportional increases in whole-blood exposure following oral or IV administration [8, 17, 18]. Following the single 100-mg dose in the present study, the geometric mean whole-blood AUC_0-24_ was 1,875 h·ng/mL and *C*_max_ was 186 ng/mL. These values approximate the steady-state exposures to ridaforolimus achieved with the therapeutic dose of 40 mg once daily for 5 days every week, but are not considered supratherapeutic. Third, higher single doses were not administered because they would not have provided proportionally higher exposure given the nonlinear blood pharmacokinetics of ridaforolimus.

Adverse events observed in this study were consistent with the known safety profile of ridaforolimus in other clinical studies, as well as the safety profiles of other mTOR inhibitors. The most common treatment-related AEs included thrombocytopenia, stomatitis, mucosal inflammation, fatigue, and diarrhea. To date, preclinical studies with ridaforolimus, as well as the safety database for ridaforolimus in cancer patients, have shown no signal for QTc prolongation. Results from this study are consistent with a preclinical risk assessment made on the basis of current ICH S7A and S7B guidelines [1]. The hERG channel was only minimally inhibited at the highest ridaforolimus concentration tested (5 % inhibition at 50 μM). This concentration for minimal hERG inhibition is more than 100 times higher than the *C*_max_ measured in whole blood following the 100-mg dose or 40-mg therapeutic regimen. The present data with a single 100-mg oral dose indicate that ridaforolimus has a low likelihood of causing delayed ventricular repolarization. Because exposure to ridaforolimus after this single dose approximates the exposure at steady-state with the 40-mg therapeutic regimen, treatment with ridaforolimus is not likely to cause a clinically meaningful prolongation of the QTc interval in patients with cancer.

## Electronic supplementary material

Below is the link to the electronic supplementary material.

Supplementary material 1 (PDF 11 kb)
