# Supplementary material for: A single supratherapeutic dose of ridaforolimus does not prolong the QTc interval in patients with advanced cancer
Source: Cancer Chemother Pharmacol. 2012 Aug 10;70(4):567–74. doi: 10.1007/s00280-012-1942-7 (PMC3456920; doi:10.1007/s00280-012-1942-7)
Supplement: Supplementary file 1 — Supplementary material 1 (PDF 11 kb) [file 280_2012_1942_MOESM1_ESM.pdf]

Electronic Supplementary Material

Article title: A Single Supratherapeutic Dose of Ridaforolimus Does Not Prolong the QTc Interval in Patients With Advanced Cancer

Journal name: *Cancer Chemotherapy and Pharmacology*

Author names: Richard M. Lush, Amita Patnaik, Daniel Sullivan, Kyriakos P. Papadopoulos, Michele Trucksis, Jacqueline McCrea, Kristine Cerchio, Xiaodong Li, Mark Stroh, Diana Selverian, Keith Orford, Scot Ebbinghaus, Nancy Agrawal, Marian Iwamoto, John A. Wagner, and Anthony Tolcher

Affiliation and e-mail address of the corresponding author: H. Lee Moffitt Cancer Center; Richard.Lush@moffitt.org

Online Resource 1. Initial QTcF interval, QTcF change from baseline, and QTcF change from baseline difference (ridaforolimus – placebo) after oral administration of placebo or single 100-mg dose of ridaforolimus in advanced cancer patients

Treatment	Hour	Number of patients[a]	Original mean QTcF value (95% CI)	Mean QTcF change from baseline (95% CI)	Difference in mean QTcF change (90% CI)
Placebo	Baseline	22	419.23 (412.61, 425.86)		
	0.5	22	420.65 (414.03, 427.28)	1.42 (-1.89, 4.74)	
	1	22	421.63 (415.00, 428.25)	2.40 (-0.92, 5.71)	
	2	21	421.68 (415.03, 428.33)	2.40 (-0.97, 5.77)	
	3	21	422.22 (415.58, 428.87)	2.94 (-0.42, 6.31)	
	4	22	420.55 (413.93, 427.18)	1.32 (-1.99, 4.64)	
	6	22	415.86 (409.24, 422.49)	-3.37 (-6.68, -0.06)	
	8	22	413.96 (407.34, 420.59)	-5.27 (-8.58, -1.96)	
	10	22	414.23 (407.60, 420.85)	-5.00 (-8.32, -1.69)	
	24	20	418.50 (411.82, 425.17)	-0.74 (-4.17, 2.69)	
Ridaforolimus (100 mg)	Baseline	20	418.08 (411.40, 424.75)		
	0.5	20	419.98 (413.30, 426.65)	1.90 (-1.53, 5.33)	0.48 (-2.80, 3.76)

1	20	419.93 (413.25, 426.60)	1.85 (-1.58, 5.28)	-0.54 (-3.83, 2.74)
2	20	419.30 (412.62, 425.97)	1.22 (-2.21, 4.65)	-1.18 (-4.50, 2.14)
3	20	421.49 (414.81, 428.16)	3.41 (-0.02, 6.84)	0.47 (-2.85, 3.79)
4	20	420.58 (413.90, 427.26)	2.51 (-0.92, 5.94)	1.18 (-2.10, 4.47)
6	20	417.20 (410.52, 423.88)	-0.87 (-4.30, 2.56)	2.49 (-0.79, 5.78)
8	20	415.29 (408.61, 421.96)	-2.79 (-6.22, 0.64)	2.48 (-0.80, 5.76)
10	20	416.96 (410.28, 423.63)	-1.12 (-4.55, 2.31)	3.89 (0.60, 7.17)
24	20	415.84 (409.16, 422.51)	-2.24 (-5.67, 1.19)	-1.50 (-4.85, 1.86)

[a]For placebo, one patient was missing data at the 2 and 3 hour time points, two patients were missing data at the 24 hour time point, and one patient’s QTcF data could not be measured. For ridaforolimus, one patient discontinued prior to day 2, one patient’s QTcF data could not be measured, and one patient was missing data and was also on a protocol violating medication.
